# Supplementary material for: HsGA20ox1, HsGA3ox1, and HsGA2ox1 Are Involved in Endogenous Gibberellin Regulation Within Heracleum sosnowskyi Ovaries After Gibberellin A3 Treatment
Source: Int J Mol Sci. 2025 May 8;26(10):4480. doi: 10.3390/ijms26104480 (PMC12110908; doi:10.3390/ijms26104480)
Supplement: Supplementary file 1 [file ijms-26-04480-s001.zip › ijms-3610882-supplementary.pdf]

Table S1. The sequences of primers used in the work.

Gene Locus	Gene Name	Forward Primer Sequence (5′ → 3′)	Reverse Primer Sequence (5′ → 3′)	Product Length (bp)
	HsGA20ox1	CTTGGCGATCAGTTTGGGAG	GTTAGGGCAGTCGGATCAGT	150
	HsGA3ox1	AAGATATTTCATGGGCCGGC	TCAACGGGAGGGACACTAAC	214
	HsGA2ox1	ATTATCCGCCATCCCCAGAT	GCCAAAACCCTATGCTTCACA	232
AB010922	*CsActin*	TTCTGGTGATGGTGTGAGTC	GGCAGTGGTGGTGAACATG	151

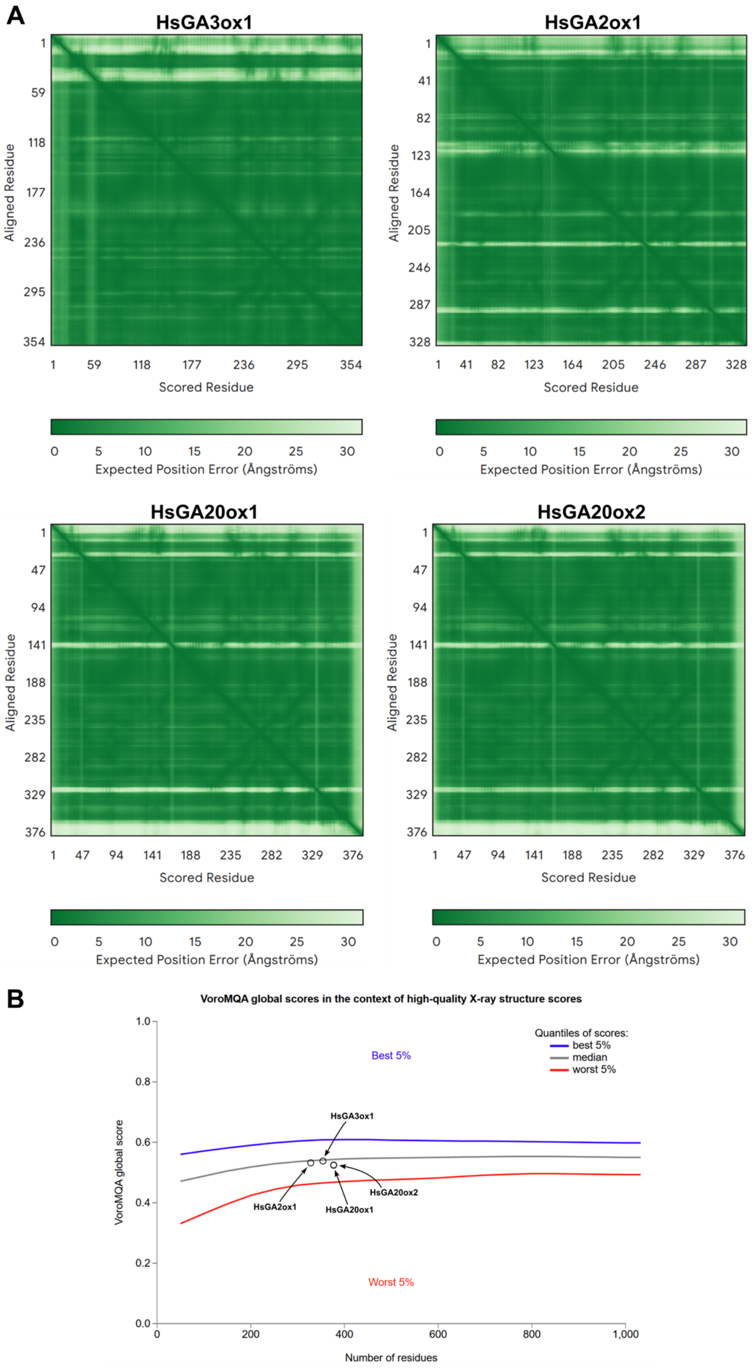

Figure S1. HsGAoxs predicted protein model structures quality assessment results from PAE (A) and VoroMQA (B).
